# Valproate prescription to women of childbearing age in English primary care: repeated cross-sectional analyses and retrospective cohort study

**DOI:** 10.1186/s12884-021-04351-x

**Published:** 2022-01-27

**Authors:** Mariangela Gaudio, Emmanouela Konstantara, Mark Joy, Jeremy van Vlymen, Simon de Lusignan

**Affiliations:** 1 Department of Clinical & Experimental Medicine, University of Surrey, Leggett Building Daphne Jackson Rd, Guildford, GU2 7XP UK; 2 Faculty of Medicine, Vita-Salute San Raffaele University, Via Olgettina 58, 20132 Milan, Italy; 3 Department of Diabetes, Faculty of Life Course Sciences & Medicine King’s College London, 10 Cutcombe Road, London, SE5 9RJ UK; 4 Royal College of General Practitioners (RCGP) Research and Surveillance Centre (RSC), 30 Euston Square, London, NW1 2FB UK; 5 Nuffield Department of Primary Care Health Medical Sciences Division, University of Oxford, Oxford, OX2 6ED UK

**Keywords:** Medical record systems, Computerised, General practice, Valproic Acid, Pregnancy, High-Risk, Contraception, Prenatal Care, Epilepsy, Bipolar Disorder, Migraine Disorders

## Abstract

**Background:**

Valproate is a teratogenic drug that should be avoided during the preconception period and pregnancy. The aim was to explore general practitioners’ (GPs) prescription patterns over time, describe trends, and explore inter-practice variation within primary care.

**Methods:**

We identified women of childbearing age (12–46 years old) in the Royal College of General Practitioners (RCGP) Research and Surveillance Centre (RSC) sentinel network. We performed repeated cross-sectional analyses from 2004 to 2018 to determine rates of prescription and a retrospective cohort estimated the prevalence of use of valproate during pregnancy.

**Results:**

In 2004, 0.31% (95% Confidence Interval (95%CI):0.18 to 0.44%) women were prescribed valproate, decreasing to 0.16% (95%CI:0.07 to 0.24%) by 2018. Among women with epilepsy, the rate fell from 15.2% (95%CI:14.4 to 16.0%) to 8.8% (95% CI:8.2 to 9.7%) over the same period. In 2018, almost two thirds (62.2%) of women who were prescribed valproate had epilepsy only, whereas bipolar disorder and migraine accounted for 15.8% and 7.4% respectively. Contraceptive prescriptions did not increase over time, and only in 2018 was there greater odds of being prescribed contraception (OR 1.41, 95%CI:1.08 to 1.45). Just under a fifth (19.7%) of women were prescribed valproate during their pregnancy; two out of three of these pregnancies were preceded by folic acid prescription (5 mg). While some practices reduced their rate of valproate prescription, others did not.

**Conclusions:**

Regulatory guidelines have changed GPs' prescription patterns in women of childbearing potential for valproate but not for contraception. Further research is needed to identify the barriers of GPs and women of childbearing potential to undertaking contraception.

**Supplementary Information:**

The online version contains supplementary material available at 10.1186/s12884-021-04351-x.

## Background

In the UK there have been a series of initiatives to curb valproate prescriptions for women of childbearing age in order to reduce pregnancy associated risks. In 2011, the National Health Service (NHS) introduced new pay-for-performance (P4P) indicators for contraception counselling for women of childbearing age with epilepsy, as part of the Quality and Outcomes Framework (QOF). Few years later, the Medicines and Healthcare products Regulatory Agency (MHRA) released a “toolkit” for patient use to raise the awareness of potential effects of valproate in pregnancy. This advised health care professionals (HCPs) to discuss the need for contraception with patients and to provide a detailed description of the risk of congenital malformations in case of pregnancy [1]. Pregnancy planning involves risk-assessment of valproate use, prescription of folic acid, and discontinuation or switch of therapy to alternative antiepileptic medication. In the event of pregnancy, urgent consultation is mandatory. In 2018, a new valproate Pregnancy Prevention Programme (PPP) was released by MHRA [2]. The PPP is directed towards both patients and HCPs and is a prerequisite for women who might become pregnant. It emphasises the necessity of highly effective contraception such as intrauterine device (IUD), implants, or sterilisation, i.e., methods with failure rates of less than 1% with typical use. Completion of an annual risk acknowledgment form is requested from the valproate user or their caregiver/guardian and from the prescribing general practitioner (GP). This is to confirm women’s understanding of the associated risks and the consequent measures which must be taken if they become pregnant while taking the drug.

Valproate is an effective medication prescribed for a variety of disorders such as epilepsy, bipolar disorder, and migraine [3–6]. In utero exposure to valproate is associated with a 10% probability of major malformations and a 30–40% probability of neurodevelopmental disorders [7, 8]. Most women of childbearing age who take potentially teratogenic medications do not use contraception, although it is strongly recommended, often due to a lack of awareness of the potential risks during pregnancy [9]. Congenital malformations (e.g. neural tube defects, spina bifida, anencephaly) have been observed in children exposed to valproate, especially during the first trimester [10, 11], while long-term neurologic and behavioural disorders including autism and learning disabilities are also well-documented [12, 13]. The risk is dose-dependent, although lower doses also come with a risk [14]. The National Institute for Health and Care Excellence (NICE) guidelines on epilepsy recommends the use of folic acid (5 mg daily) for any woman using antiepileptic drugs who is considering pregnancy, since this has been demonstrated to decrease the risk of malformations [15].

Given the adverse effects of taking valproate during pregnancy and the subsequent change in guidelines, it is important to investigate whether these are implemented in general practice. This will allow us to understand whether more education and training about the programme is needed.

### Objectives

The objectives were therefore to: describe trends in valproate prescription over time to women of childbearing age in primary care; report the pregnancy rate of women prescribed valproate; report folic acid prescription to women during a six-month preconception period as a surrogate marker for pregnancy planning; report contraception prescription; and report inter-practice variation regarding the implementation of guidelines.

## Methods

### Study design

The study design had two components:

1. Repeated cross-sectional design to report trends of the rate of prescription of valproate over time and inter-practice variability. This included all women with childbearing potential registered at any point and for a complete year when aged between 12 and 46 years old in the years of interest.
2. Retrospective cohort design to report the use of folic acid and the outcomes of pregnancy for women exposed to valproate during the preconception period. This comprised all women who had at least one pregnancy and prescription of valproate.

### Setting

The Royal College of General Practitioners (RCGP) Research and Surveillance Centre (RSC) is a sentinel network which collects data from more than 250 general practices across England. At the end of December 2018, 2,901,728 patients were registered to RCGP RSC, representing 4.5% of the national population (compared to Overview of the UK population—Office for National Statistics). The population is slightly skewed towards younger, living in London and urban locations, more ethnically mixed and less deprived – though these differences are small [16]. The database grew larger as more practices joined the network over the period of the study (Supplementary files, Supplementary Table 1). All RCGP RSC data are pseudonymised at the point of data extraction and no personally identifiable data are available to researchers.

Computerised medical records allow capturing of routine diagnostic and management data, including nearly all prescriptions. Since the introduction of QOF for chronic disease management, data on long-term conditions are more complete. Two of the conditions for which valproate is prescribed (epilepsy and bipolar disorder) are included in the financially incentivised QOF indicators; bipolar disorder is included in the mental health indicator. For these reasons, the RCGP RSC is well placed to identify prescription trends of valproate, folic acid, and contraception in general practice.

### Key variables

Demographic and clinical data included: age, ethnicity, socioeconomic status (using Index of Multiple Deprivation—IMD); smoking status (current, former, or non-smoker), valproate prescription, and diseases for which valproate is indicated (epilepsy, bipolar disorder, and migraine).

We extracted hormonal contraception prescription data: oral contraceptive pill (OCP), progesterone only pill (POP), injectable contraceptive and implants, and intrauterine device (IUD). Use of IUDs was presumed for 5 years after the first date its prescription. We captured information about pregnancy using an ontological approach. An ontology is a classification of concepts, used in this study to record pregnancies in computerised medical record (CMR) systems. The pregnancy ontology looks for codes associated with the start of pregnancy and codes reporting whether pregnancy ran to term. Reasons that pregnancies did not reach term included spontaneous or induced abortion, or stillbirth. The ontology also has a temporal element associating start and end of pregnancy codes within a year of each other [17]. Hence, we captured data about folic acid prescription in the 6 months before and during pregnancy. We also made a temporal association between valproate prescription and pregnancy. We looked for the valproate prescription immediately before the first code indicating the start of pregnancy (within 6 months as pregnant women may not present to their GP until pregnancy is established) and during pregnancy.

Practices were categorised by size based on patient numbers (small, medium, and large).

### Statistical methods

We performed repeated cross-sectional analyses to describe the trend in valproate prescription over fourteen years (2004–2018). We used descriptive statistics to summarise the demographics of the study population. We reported the rate of valproate prescription as a percentage with 95% confidence intervals (95% CI). We ordered reasons for valproate prescription into a hierarchy of importance—epilepsy as most important, followed by bipolar disorder, then migraine. For example, a woman who was prescribed valproate due to epilepsy and migraine would only appear in the epilepsy group.

We reported contraception data for women who were not pregnant during the years of interest. A woman was described as a ‘contraception user’ or ‘not at risk of pregnancy’ if she had been prescribed contraceptives or had a record of sterility/infertility. Odds ratios were calculated using multivariable logistic regression models whilst adjusting for age, ethnicity, IMD, smoking habit, and comorbidities.

In order to explore inter-practice variation for the study period, we created funnel plots [18], by plotting general practices’ prescription rates against their registered practice’s size. Diffusion of Innovation Theory was used to identify a practice’s uptake of valproate PPP [19]. We took prescription rates and compared them to practice size in both 2004 and 2018 and mapped them onto the Diffusion of Innovation model: in 2018, practices whose prescription rates lay above 2–3 standard deviations from the mean were interpreted as “laggards”, or lagging behind policy.

Statistical analyses were carried out using RStudio, version 3.5.3. The manuscript follows STrengthening the Reporting of OBservational studies in Epidemiology (STROBE) guidelines.

## Results

### Demographics & Trends

**Fig. 1:**
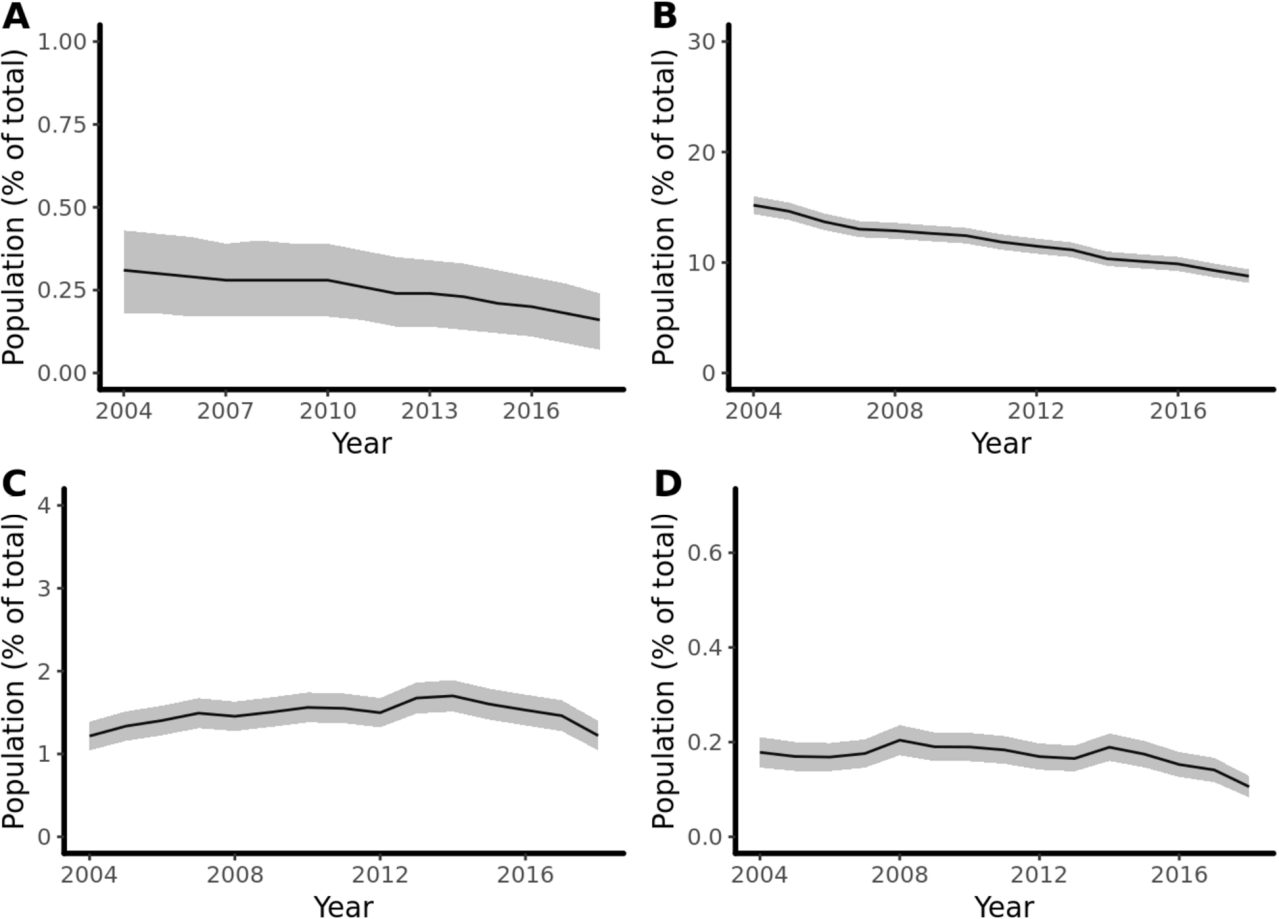
Trends over time of proportions of women of childbearing age prescribed valproate in RCGP RSC. Legend: A) Prescription of valproate among all women of childbearing age registered each year to the RCGP RSC; B) Prescription among women of childbearing age with epilepsy; C) Prescription among women of childbearing age with bipolar disorder; D) Prescription of valproate among women of childbearing age with or migraine

We identified 533,627 women of childbearing age in 2004 and 729,662 in 2018. In 2004, 0.31% (95%CI: 0.18 to 0.44%; 1,639/ 533,627) of women of childbearing age were prescribed valproate. In 2018 the rate fell to 0.16%, (95%CI: 0.07 to 0.24%; 1,149/729,662), a fall of 48.7% over 15 years (Fig. 1A). Males of the same age group were prescribed valproate consistently over the same period from 0.37% (95%CI: 0.35 to 0.38%) to 0.36% (95%CI: 0.34 to 0.37%).

Figure 1 shows trends of prescription rates for specific indications. In both 2004 and 2018, valproate was most commonly prescribed in epilepsy [2004: 15.2% (95% CI: 14.4–16.0%); 2018: 8.8% (95%CI: 8.2 to 9.4%)], followed by bipolar [2004: *n* = 1.2% (95% CI: 1.0–1.4%); 2014: 1.7% (95%CI: 1.5 to 1.9%); 2018: *n* = 1.2% (95% CI: 1.0 to 1.4)] and then migraine [2004: 0.18% (95% CI: 0.15 to 0.21%); 2018: 0.11% (95% CI: 0.08 to 0.13%)]. Only a small number of women were prescribed valproate for multiple diagnoses. Demographic changes can also be observed between 2004 and 2018 (Table 1). Women prescribed valproate in 2018 were older than those in 2004.

**Table 1:** Descriptive statistics for women of childbearing age prescribed valproate in 2004 and 2018

	**Year *n* (%)**		
	2004	2018	Chi-square
** N**	1639	1149	P value
**Demographics**			
** Age range**			0.001
** Under 20**	158 (9.6)	133 (11.6)	
** 20–29**	378 (23.1)	238 (20.7)	
** 30–39**	607 (37.0)	366 (31.9)	
** Over 40**	496 (30.3)	412 (35.9)	
** IMD**			NS
** 1 (most deprived)**	355 (22.2)	285 (25.4)	
** 2**	322 (20.1)	224 (19.9)	
** 3**	294 (18.4)	205 (18.3)	
** 4**	332 (20.7)	213 (19.0)	
** 5 (least deprived)**	299 (18.7)	196 (17.5)	
** Ethnicity**			< 0.001
** White**	966 (58.9)	842 (73.3)	
** Asian**	48 (2.9)	62 (5.4)	
** Black**	22 (1.3)	24 (2.1)	
** Mixed**	10 (0.6)	17 (1.5)	
** Other**	4 (0.2)	8 (0.7)	
** Missing**	589 (35.9)	196 (17.1)	
** Smoking**			< 0.001
** Non-smoker**	617 (37.6)	465 (40.5)	
** Current smoker**	557 (34.0)	270 (23.5)	
** Former smoker**	233 (14.2)	316 (27.5)	
** Missing**	232 (14.2)	98 (8.5)	
**Indications**			
** Epilepsy**	1149 (70.1)	715 (62.2)	< 0.001
** Bipolar Disorder**	188 (11.5)	181 (15.8)	0.001
** Migraine**	118 (7.2)	85 (7.4)	NS
** Epilepsy and Migraine**	165 (10.1)	116 (10.1)	NS
** Epilepsy and Bipolar disorder**	46 (2.8)	30 (2.6)	NS
** Bipolar and Migraine**	60 (3.7)	54 (4.7)	NS

Data are presented as *n* (%)

Abbreviation: *IMD*, Index of Multiple Deprivation *NS*, Not significant

The prescription rate of valproate decreased in women of childbearing age consistently across all age groups. The biggest decline was observed among the group aged 20–29 years old (–59.5%), followed by those aged 30–39 years old (–54.2%). Smaller reductions were observed in women younger than 20 (–40.6%) or more than 40 years old (–28.6%) (Supplementary files, Supplementary Figure 1).

### Contraception

Contraception prescription for women of childbearing age using valproate was stable, changing little from 32.5% (95%CI: 30.2 to 34.8%) in 2004 to 33.7% (95%CI: 30.9 to 36.4%) in 2018 (Supplementary files, Supplementary Figure 2). Positive association between contraceptive provision and valproate prescription only emerged in 2018 with OR 1.41 (95%CI: 1.23 to 1.61, *p* < 0.001, ST2). Women prescribed valproate were more likely to have a prescription of progesterone only pill or injectable contraception. Sterilisation was also more common among women that were prescribed valproate (Table 2).

**Table 2:** Contraception type difference according to valproate prescription

Type of contraception	Women of childbearing age	Women of childbearing age prescribed valproate	*P* value
n	185,997	382	
OCP	91,893 (49.4)	84 (22.0)	< 0.001
POP	62,412 (33.6)	134 (35.1)	NS
Injectable	13,896 (7.5)	67 (17.5)	< 0.001
Implants	12,754 (6.9)	36 (9.4)	0.06
IUD	9,521 (5.1)	20 (5.2)	NS
Sterilisation	4,748 (2.6)	47 (12.3)	< 0.001

Data are presented as *n* (%)

Abbreviation: *OCP*, Oral Combined Pill *POP*, Progesterone only pill *IUD* Intrauterine device *NS*, Not Significant

### Pregnancy

Over 1,500 women (*n* = 1,729) who were prescribed valproate had history of at least one pregnancy (Table 3). Mean age of the first pregnancy was 28.2 years (SD 6.8, median 28, IQR 10). Of these, 335 (19.7%, 95%CI: 17.8 to 21.5%) were prescribed valproate during their pregnancy. The majority of these women were white, of lower socioeconomic status, and nearly 80% lived in urban areas. Over 100 women (*n* = 129; 38.5%) were marked as ‘current smoker’ prior to pregnancy. Most common indication for valproate prescription was epilepsy (72.2%), followed by bipolar disorder (14.0%) and migraine (3.9%). Two thirds were prescribed folic acid before and during pregnancy and in 14.9% of the cases pregnancy did not reach term.

**Table 3:** Characteristics of women using valproate during pregnancy

Women who used valproate during pregnancy *N* = 335	
Ethnicity	
White	230 (68.7)
Asian	20 (6.0)
Black	6 (1.8)
Mixed	6 (1.8)
Other	1 (0.3)
Missing	72 (21.5)
IMD	
1 (most deprived)	91 (28.4)
2	55 (17.2)
3	59 (18.4)
4	72 (22.5)
5 (least deprived)	43 (13.4)
Urban–rural	
Urban	267 (79.7)
Rural	53 (15.8)
Missing	15 (4.5)
Smoking	
Non-smoker	95 (28.4)
Current smoker	129 (38.5)
Former smoker	62 (18.5)
Missing	49 (14.6)
Epilepsy	242 (72.2)
Bipolar Disorder	47 (14.0)
Migraine	13 (3.9)
Valproate prescription within six months prior to pregnancy	247 (73.7)
Folic acid within six months prior to pregnancy	207 (61.8)
Folic acid during pregnancy	199 (59.4)
Pregnancy not to term	50 (14.9)

Data are presented as n (%)

Abbreviation: *IMD*, Index of Multiple Deprivation

### Inter-practice variation

**Fig. 2:**
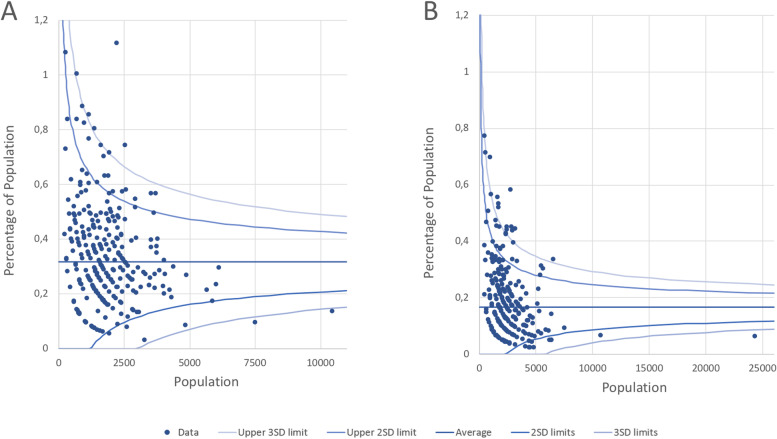
Funnel plots of valproate prescription rates to women of childbearing age among RCGP RSC practices in A) 2004 and B) 2018

Valproate prescription to women of childbearing age did not consistently decrease in all practices of the RCGP RSC, but it was characterised by significant inter-practice variability (Fig. 2). In 2004, few practices (22 out of 269) reported increased prescription of valproate compared to others (*p* < 0.025). By comparison, in 2018 more practices’ (31/260) prescription rates laid outside the upper control limit. The majority of these practices (23/31) had patients with low socioeconomic status (IMD quintiles 1–3). Nearly half of these (16/31) were large sized practices, 10 were medium, and 5 were small. We identified nine practices (3.3%) above the upper control limits in both years (laggards), and a further 22 practices (8.2%) appeared not to have changed their prescribing behaviours as other practices reduced prescribing.

## Discussion

### Main findings

Prescription of valproate to women of childbearing age declined by almost half over the 14-year study period, with the greatest decline observed in those aged 20 to 29 years old. Prescription rates decreased for all indications for which valproate is usually prescribed (epilepsy, bipolar disorder, and migraine). In 2018, women prescribed valproate were slightly more likely to receive contraception, however the difference between years was small and only one third of women taking valproate were prescribed contraception.

Of all women prescribed valproate who had a pregnancy, one fifth had a prescription during gestation. Most of them were prescribed 5 mg of folic acid.

Over the years, the majority of practices reduced their prescription of valproate, showing less variation in 2018. However, just under 10% of practices did not change their valproate prescription during the observed period. The majority of these practices were made up of patients of lower socioeconomic status.

### Comparison with other studies

Other studies in Europe reported a decrease in valproate prescription in women of childbearing age. For example, in Germany in 2017 a card was released to GPs to inform patients about valproate, and a study reported decreasing trends in prescription of valproate in women of childbearing age, especially for epilepsy [20, 21]. Similarly, an Irish study described similar trends in valproate prescription among women for epilepsy; however, in line with our findings, folic acid and contraception prescription rates were low in women prescribed valproate [22]. Furthermore, in 2017, the French National Agency for the Safety of Medicines and Health Products (ANSM) suggested that women of childbearing age with bipolar disorder or migraine who were not using contraception should not be prescribed valproate [23]. The effect of these measures is yet to be evaluated.

### Strengths and limitations of the study

Data were collected from UK general practices, which have been computerised since the 1980s. Practices have received long-term feedback about data quality, most recently via a dashboard. However, the RCGP RSC network is only able to collect coded data, meaning free-text information is missing from analyses. Furthermore, the data on contraception and folic acid may be incomplete as they can be prescribed by other healthcare providers (i.e., secondary care) and folic acid can be purchased over the counter. Finally, some women did not have a code in their records to indicate why they had been prescribed valproate; therefore, we were unable to determine the underlying condition.

### Implications for research and practice

Providing preconception care within primary practice to women of childbearing age can improve neonatal outcomes [24]. The results from this study suggest the valproate PPP have been partially successful, with a clear decline in the rate of valproate prescription over the period of this study. However, this study found very little change in prescription of contraception in women of childbearing age. It appears that some practices have been less receptive to the quality improvement interventions than others. This suggests that there is more to be done to ensure that women are receiving the best preconception and pregnancy care possible. Lessons can be learnt from other quality improvement interventions that have a higher uptake, such as the P4P scheme QOF, which has adoption rates as high a 99% [25].

Future research should focus on identifying why uptake of PPP is low and how to improve it. Assessment of the impact of the latest measures should also be repeated once sufficient time has elapsed from the adoption of PPP by GPs. With a growing body of evidence showing that valproate should be avoided – where possible – during pregnancy, prescription and contraception patterns should be quantified in women taking other medications (e.g., ACE inhibitors, lithium, statins) and with other conditions in order to identify additional perinatal risks. The development and implementation of advanced, interactive dashboards that can identify women who do not use contraception and might become pregnant could help prevent unplanned pregnancies. An example is the RCGP RSC tool “MyPracticeDashboard” which compares practices who have joined the network. A possible further development could be the introduction of a new section which identifies women of childbearing age to monitor preconception health, especially for those with high-risk pregnancies.

Qualitative research is needed to explore what other methods of contraception women use (e.g., condoms), the contraception practices in secondary care (i.e., sexual health clinics, obstetrics and gynaecology clinics, psychiatry clinics), GPs’ barriers and facilitators to prescribing high-effectiveness contraception, and the barriers and facilitators to undertaking such contraception in women of childbearing age who use valproate and other potentially harmful medications.

## Conclusions

Prescription of valproate has steadily decreased in general practice since 2004, however, contraception prescription has not increased. While a range of interventions have been attempted, it is possible that a well-rewarded QOF indicator could build on the work of the UK’s medical regulator to achieve greater impact.

## Supplementary Information

**Additional file 1:** **Supplementary Table 1.** Women of childbearing age of RCGP RSC cohort registered each year. **Supplementary Figure 1.** Prescription of valproate to women of childbearing age in the general population divided in four age groups. **Supplementary Figure 2. **Trends over time of contraception prescription to women of childbearing age using valproate. **Supplementary Table 2.** Adjusted odds ratios of contraception patterns among pathologies and valproate prescription. Covariates adjusted for: age; deprivation quintile; smoking status; epilepsy; bipolar disease; migraine.

## Data Availability

The datasets generated for this study can be found in the RCGP RSC repository. RCGP RSC data are available for the analysis following the application form that can be found online at www. rcgp.org.uk/rsc.

## Abbreviations

GPs: General practitioners’
NHS: National Health Service
RCGP: Royal College of General Practitioners
RSC: Research and Surveillance Centre
P4P: Pay-for-performance
QOF: Quality and Outcomes Framework
MHRA: Medicines and Healthcare products Regulatory Agency
HCPs: Health care professionals
PPP: Pregnancy Prevention Programme
NICE: National Institute for Health and Care Excellence
IMD: Index of Multiple Deprivation
OCP: Oral contraceptive pill
POP: Progesterone only pill
IUD: Intrauterine device
CMR: Computerised medical record
STROBE: STrengthening the Reporting of OBservational studies in Epidemiology
