# Supplementary material for: Valproate prescription to women of childbearing age in English primary care: repeated cross-sectional analyses and retrospective cohort study
Source: BMC Pregnancy Childbirth. 2022 Jan 27;22:73. doi: 10.1186/s12884-021-04351-x (PMC8793222; doi:10.1186/s12884-021-04351-x)
Supplement: Supplementary file 1 — Additional file 1: Supplementary Table 1. Women of childbearing age of RCGP RSC cohort registered each year. Supplementary Figure 1. Prescription of valproate to women of childbearing age in the general population divided in four age groups. Supplementary Figure 2. Trends over time of contraception prescription to women of childbearing age using valproate. Supplementary Table 2. Adjusted odds ratios of contraception patterns among pathologies and valproate prescription. Covariates adjusted for: age; deprivation quintile; smoking status; epilepsy; bipolar disease; migraine. [file 12884_2021_4351_MOESM1_ESM.docx]

**Supplementary Files**

**ST1**: Women of childbearing age of RCGP RSC cohort registered each year.

| Year | Denominator |
| --- | --- |
| **2004** | 502,783 |
| **2005** | 535,266 |
| **2006** | 555,876 |
| **2007** | 574,413 |
| **2008** | 589,540 |
| **2009** | 598,973 |
| **2010** | 609,125 |
| **2011** | 623,279 |
| **2012** | 638,295 |
| **2013** | 654,442 |
| **2014** | 674,969 |
| **2015** | 693,886 |
| **2016** | 702,854 |
| **2017** | 574,413 |
| **2018** | 589,540 |

**SF1:** Prescription of valproate to women of childbearing age in the general population divided in four age groups.


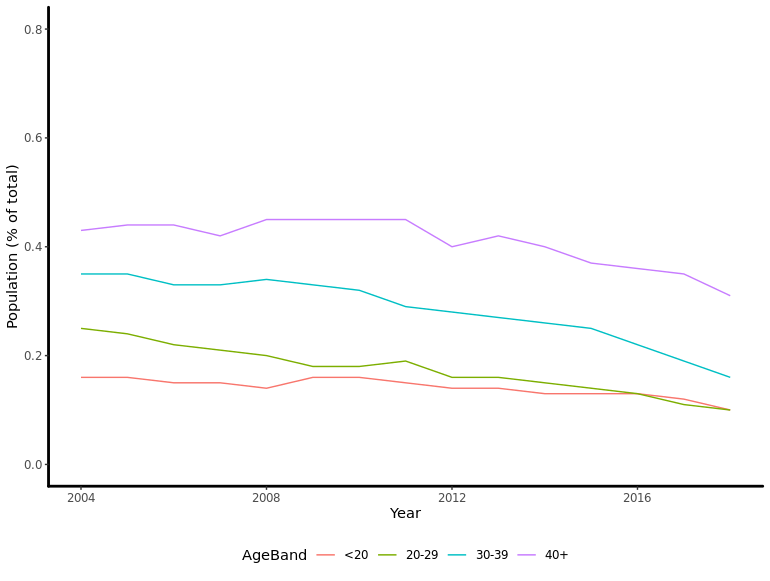


**SF2:** Trends over time of contraception prescription to women of childbearing age using valproate.


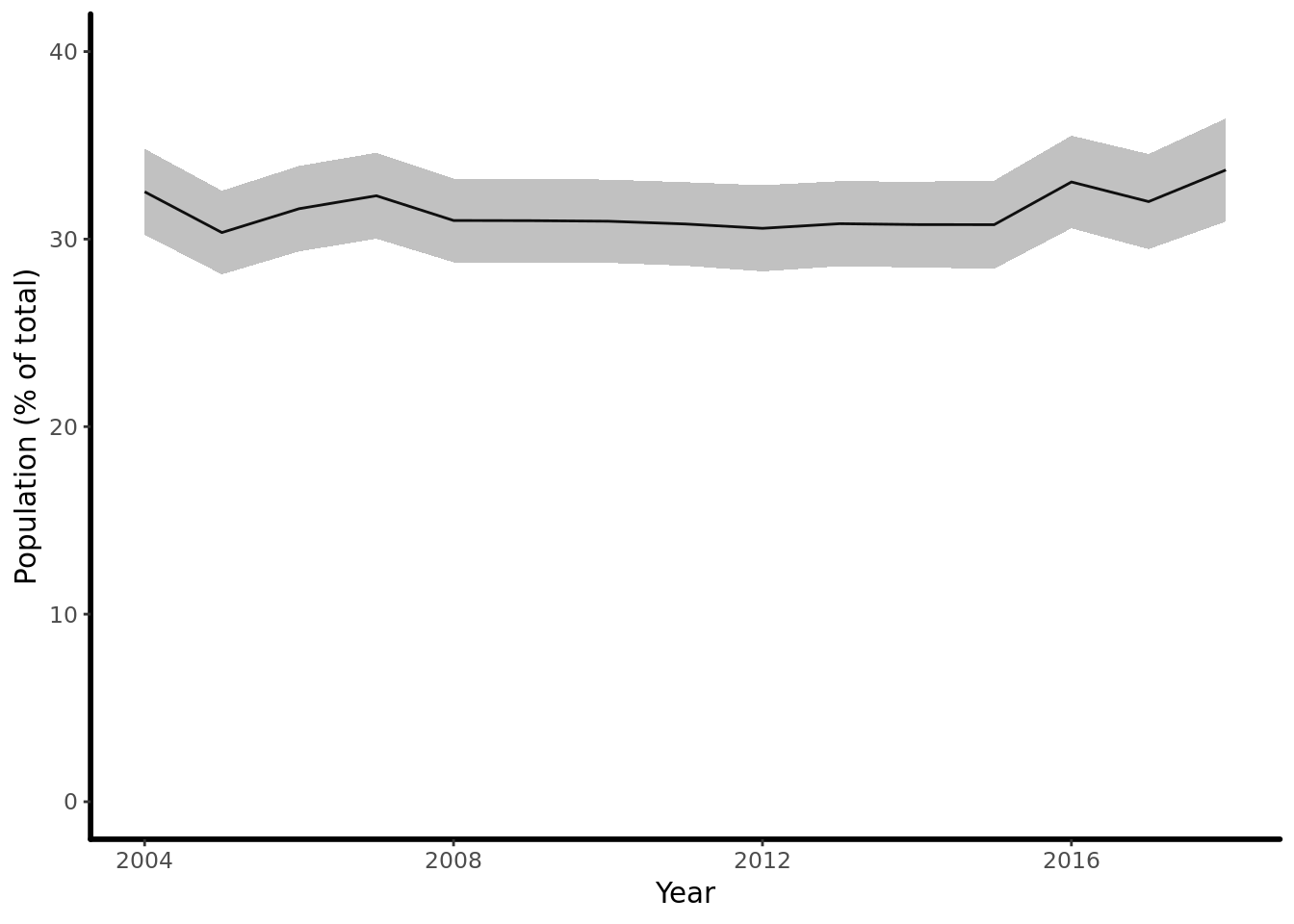


**ST2:** Adjusted odds ratios of contraception patterns among pathologies and valproate prescription. Covariates adjusted for: age; deprivation quintile; smoking status; epilepsy; bipolar disease; migraine.

| **Association with contraception** | **Odds Ratio** | **95%CI** | **P value** |
| --- | --- | --- | --- |
| **Women prescribed valproate** | 1.41 | (1.23-1.61) | <0.001 |
